# Augmented reality books: in-depth insights into children’s reading engagement

**DOI:** 10.3389/fpsyg.2024.1423163

**Published:** 2024-08-14

**Authors:** Kawla Alhamad, Andrew Manches, Sarah McGeown

**Affiliations:** ^1^Moray House School of Education and Sport, University of Edinburgh, Edinburgh, United Kingdom; ^2^College of Education, Imam Abdulrahman Bin Faisal University, Dammam, Saudi Arabia

**Keywords:** reading, engagement, augmented reality, books, children

## Abstract

Children’s reading engagement is associated with the quality of their reading experiences and outcomes; however, research to date has only examined children’s reading engagement within the context of traditional print books or digital texts. Augmented Reality represents a hybrid reading experience, where traditional paper books are augmented with digital features (e.g., animations, sounds, comprehension questions). This is the first study to examine children’s perspectives and experiences of AR books, within the context of reading engagement. In total, 38 demographically diverse children (aged 8–10, 21 male, 17 English as an Additional Language, 14 ethnicities, nine with teacher-reported reading difficulties) from the UK participated. After reading an AR book, children participated in interviews about their reading engagement. Deductive (themes) and inductive (subthemes) approaches to thematic analysis were used, examining children’s AR reading experiences within the context of their behavioral, cognitive, affective and social engagement. The majority of children found AR books easy to use, and provided examples of how AR books supported their behavioral engagement (e.g., desire to read more/extend reading practices), altered their cognitive engagement (e.g., reading strategies, visual representation/use of imagination, comprehension monitoring), influenced their affective engagement: (e.g., diverse positive feelings), and social engagement (e.g., prompted interaction and discussion), providing examples suggesting similarities and differences with traditional print books. This paper provides novel in-depth insights into children’s perspectives and experiences of AR books, and provides a foundation for researchers, educators, and AR book designers interested in better supporting children’s reading experiences and outcomes with AR books.

## Introduction

Over the last two decades, children’s reading practices have shifted from the almost exclusive use of paper-based texts to an increasing use of digital devices, as children spend more time engaging in diverse literacy activities (Wang et al., 2019; Halamish and Elbaz, 2020; Furenes et al., 2021; Polyzou et al., 2023). Although little researched, Augmented Reality (AR) is an emerging technology which offers child readers a novel reading experience (Wang et al., 2019; Danaei et al., 2020) as it combines both print and digital forms. Whilst educational claims surrounding new technologies need to be approached cautiously, combining print and digital media does raise interesting questions about the nature of children’s reading experiences, as well as important pedagogical and design opportunities for those wishing to support children’s reading. To date, research has explored the potential value of AR in educational settings (e.g., Bujak et al., 2013; Cuendet et al., 2013; Wu et al., 2013; Akçayır and Akçayır, 2017; Chen et al., 2017; Fernández-Batanero et al., 2022), for example within AR games (Tobar-Muñoz et al., 2017), AR-based learning materials (Chien et al., 2019), AR applications (Shaaban and Mohamed, 2024) and AR books (Liu et al., 2023). However, no research has been conducted to explore how AR books shape children’s reading engagement, from *their* perspectives. This paper therefore aims to contribute novel insights into children’s perspectives and experiences of using AR books, examining if, and how, AR books influence their reading engagement.

### Augmented reality books

**Figure 1:**
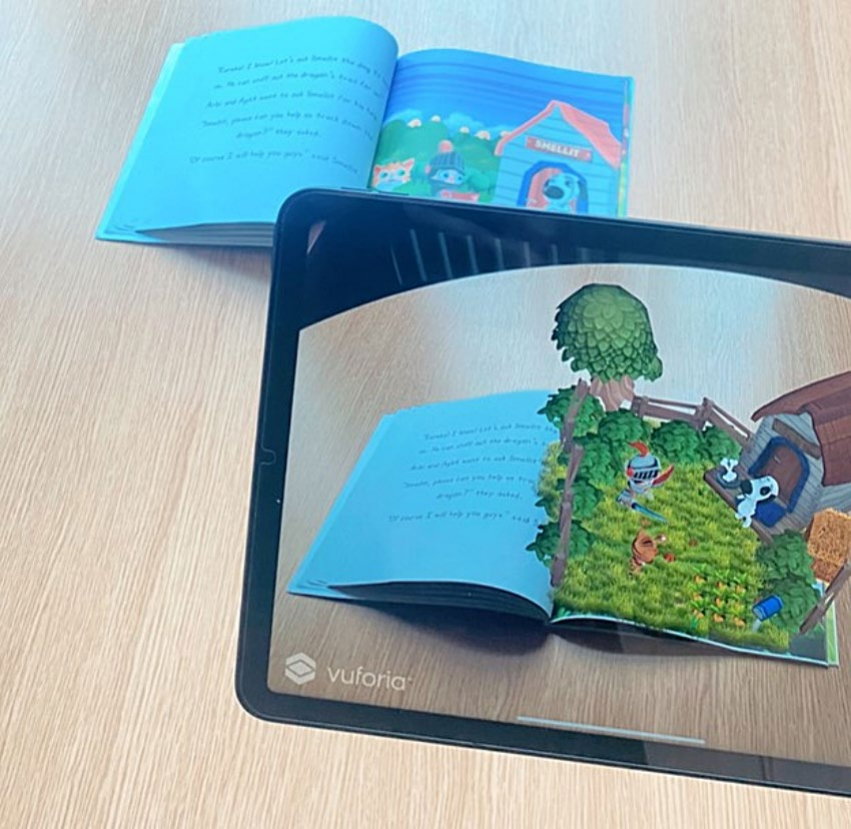
Augmented reality book.

Augmented Reality (AR) technology is increasingly being integrated into educational settings to support students’ learning (e.g., Kerawalla et al., 2006; Phadung et al., 2017), and augmented reality books represent one of its recent implementations (Panchenko et al., 2020). While there are various and evolving instantiations of Augmented Reality, the term generally refers to the superimposition of digital information on the physical world, typically via a screen device (see Figure 1). In the context of AR books, this enables traditional paper books to be enhanced through the addition of AR features such as sounds, 3D animations, and interactive questions (Yilmaz et al., 2017; Wang et al., 2019; Danaei et al., 2020). AR books therefore invite more physically interactive reading experiences as students explore and combine paper and virtual content (Wang et al., 2019; Danaei et al., 2020).

The enhancement of paper books is not new, for example, search and find activities, tactile features such as pop-ups and flaps, and comprehension questions within children’s books are all common features (Vanderschantz et al., 2019). AR however allows a digital extension of this, for example, to include sounds, animations, graphics (Vanderschantz et al., 2019; Besa, 2021; Shinskey, 2021) and feedback on comprehension questions. As a result of these technical affordances, it has been claimed that AR technology can positively affect students’ reading attitudes and motivation (e.g., Yilmaz et al., 2017; Othman et al., 2021; Roumba and Nicolaidou, 2022), in addition to supporting reading concentration (Wang et al., 2019), comprehension (Dünser and Hornecker, 2007; Danaei et al., 2020), story retelling (Liu et al., 2023), learning effectiveness (Chang et al., 2023) and transmission of knowledge (Fernandes and Leite, 2024).

Given the relatively recent emergence of AR and the complexity of factors shaping if and how children interact with AR books, qualitative research is necessary to better understand children’s perspectives and experiences of AR books and how it relates to their reading engagement. Compared to traditional print books, readers can engage with AR books in different ways. For example, research with digital books highlights different navigation profiles by both child and young adult readers (Javorsky and Trainin, 2014; Turner et al., 2020). In addition, Zhou and Yadav (2017) found that young children’s behavioral and affective reading engagement were positively affected by reading multimedia stories compared with paper-based storybooks. Similarly, Moody et al. (2010) noted that children who participated in an adult-led e-storybook session had a higher persistence level than those who participated in an adult-led traditional storybook session. Different navigation profiles, or ways in which readers engage with AR books, can therefore have consequences for their reading experiences and/or outcomes. Yet there is an absence of research exploring children’s perspectives and experiences of AR books and how they perceive it to support, or impair, their reading engagement.

### Reading engagement

**Figure 2:**
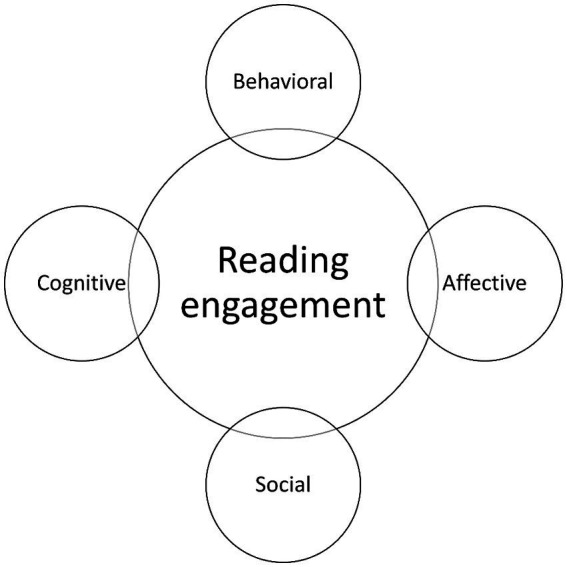
Reading engagement framework. Adapted with permission from McGeown and Smith (2024), original framework proposed by Lee et al. (2021).

Until recently, research into children’s reading engagement has been hindered by a lack of conceptual and operational clarity (Unrau and Quirk, 2014; Lee et al., 2021). Indeed, researchers understanding of reading engagement has evolved considerably over the last decade, from a two (affective and behavioral) to three (affective, behavioral and cognitive) (Fredricks et al., 2004; Unrau and Quirk, 2014; Barber and Klauda, 2020; Cao et al., 2024; Clinton-Lisell et al., 2024) dimensional construct. In different studies, reading engagement has been conceptualized in different ways, and has been explored within the context of different reading practices (e.g., independent or shared reading) and/or with different text types (e.g., traditional print or digital books: see Clinton-Lisell et al., 2024 for a review). In a recent systematic review of reading engagement research (Lee et al., 2021) four dimensions of engagement were identified: behavioral, cognitive, affective and social (see also McGeown and Smith, 2024) (see Figure 2). While previous research has drawn upon these different dimensions of engagement to understand the relationship between children’s reading experiences and outcomes (e.g., Miyamoto et al., 2019; Torppa et al., 2020; Clinton-Lisell et al., 2024), these four dimensions have not yet been studied in parallel. Behavioral engagement reflects children’s reading behaviors, for example, the frequency and duration of children’s reading, the way in which they read, and the nature of the text types read. Research demonstrates that more time spent reading, specifically book reading, and specifically fiction book reading, leads to better reading and language skills (Torppa et al., 2020; Van Bergen et al., 2021; Nation et al., 2022). Cognitive engagement reflects children’s level of cognitive effort while reading, and the application of goal-directed cognitive strategies (e.g., re-reading, decoding, drawing upon background knowledge) to support their comprehension. Indeed, research demonstrates that metacognitive knowledge of strategy use has been found to mediate the relationship between children’s reading motivation and skill (Miyamoto et al., 2019). Affective engagement reflects the breadth and depth of diverse emotions and feelings experienced by children while reading, for example enjoyment, interest, anticipation, excitement, sadness, etc. Research demonstrates that positive reading experiences are essential for children to choose to read more in future, but that even stories which elicit negative emotions, e.g., sadness, can be reported by children as enjoyable (McGeown and Wilkinson, 2021; Clark et al., 2023; Currie and McGeown, 2024). Finally, social engagement refers to children’s participation in reading activities with others, as they read together, share, swap, and discuss books they have read. Social reading activities includes both agentic and non-agentic interactions (Lee et al., 2021), and when positively perceived and experienced, can foster enjoyable and engaging reading experiences (Cremin, 2014; McGeown and Wilkinson, 2021).

This framework of reading engagement offers a comprehensive approach to study and understand children’s experiences with AR books, given the technology’s potential to alter or extend children’s reading engagement in ways which have not been previously studied in traditional print books, nor with digital texts. For example, AR books are likely to have consequences for children’s behavioral engagement, inviting more interactive reading behaviors, as children use a digital device to switch between the text, illustrations and AR features, and engage with the AR digital content (Dünser and Hornecker, 2007; Fiusa et al., 2023). AR books are also likely to influence children’s cognitive strategies while reading (Cheng, 2016), as they are required to integrate content from the text, illustrations and the AR features to comprehend the story, with AR features offering an additional source of information to support (or hinder) comprehension. It is further possible that AR books invoke new affective reading experiences, given their distinctly different nature when compared to traditional printed books, and also the probability that they are novel for most children. Finally, AR books may also shape social interactions, where the integration of physical and digital interactivity creates new opportunities for children to explore and discuss AR books together (Matcha and Rambli, 2012).

### Research rationale and aim

Children’s reading enjoyment and engagement are at an all-time low (Clark et al., 2023), yet digital literacy practices are becoming increasingly present in children’s lives (Picton et al., 2022). AR books offer a way to blend traditional and digital forms of reading, yet there is an absence of research exploring children’s perspectives and experiences with AR books. As the ultimate users of AR books, children’s thoughts and insights are essential to inform educators, designers and authors to optimally improve children’s reading experiences and outcomes, in addition to identifying new questions for future research. This study aims to explore children’s perspectives and experiences of Augmented Reality (AR) books, within the framework of reading engagement, and seeks to address two research questions as follows:

1. How do children read/interact with an AR story book in a classroom setting in real-time?
2. To what extent, and how, do children perceive the features of an AR story book as supportive of their reading engagement?

## Materials and methods

### Participants

A demographically diverse sample of 38 (21 boys, 17 girls) children (aged 8–10) the UK participated in this study. Participating children represented 14 different nationalities, with 17 speaking English as an Additional Language (EAL). In addition, nine had teacher-reported reading difficulties (seven in comprehension and two in decoding) and one student had a genetic learning disability. Self-reported levels of reading enjoyment were requested and 16 children reported high, 16 medium/intermediate, and 6 low levels of reading enjoyment. Following ethical approval from Moray House School of Education and Sport, University of Edinburgh, headteacher, parent/guardian and child consent was obtained prior to participation.

### Procedure

Prior to the study, all children participated in a short workshop in their classroom, lasting approximately 15 min, to learn what AR books are and to see a demonstration of how AR books work. Children then participated in an AR book reading session which took place in a quiet space in the school library and lasted approximately 30 min, with approximately 15 min reading the AR book and 15 min answering interview questions. Students were observed interacting naturally with the AR book (printed book, with iPad affording AR features), with the support of the researcher if required. Written (anonymized) notes were made in relation to usability/accessibility, which was defined as whether the reader demonstrated easy engagement with the AR book based on observable behaviors, including managing alternation between the book and the iPad, activating images via iPad, and responding to the interactive questions. Accessibility was determined based on how many times the reader received help from the researcher to manage the alternation, the images’ activation, and the interactive questions. Children were given the option to read the AR book alone or in a dyad (dyad pairs were chosen by teacher), with the researcher present in all sessions. Following this, the interview questions explored the four dimensions of reading engagement described earlier (behavioral, cognitive, affective and social). In total, twenty-two sessions were conducted (6 alone, 16 dyads). All interviews were audio recorded. Aligned with open research practices, the study was preregistered and can be accessed here: https://osf.io/9q678.

### The AR storybook

Using online bookstore catalogs, AR books were reviewed to find a book suitable for the age group of the study. Following agreement of the research team, Arbi 1 (Burguera, 2015) (https://www.arbibook.com) was selected due to the simplicity of AR interaction (device held over full-page image), suitability of language/content for age range, genre (fiction), length (appropriate for assessment sessions), and quality of story, illustrations, and AR features. The book was written by Iker Burguera and published in 2015 by CreateSpace Independent publishing platform. The Arbi 1 App was downloaded from the App Store to access the AR features through the iPad. The theme of the Arbi 1 story is around friendship, where friends with different abilities work together to protect their village from a dragon. The book contained 40 pages (16 pages of text on left hand and 16 pages of full-page illustration on right hand), with additional pages providing general information and instructions.

### Data analysis

All interviews were audio recorded and themes were identified using a combination of deductive (four dimensions of engagement: behavioral cognitive, affective, social) and data-driven inductive (subthemes) approaches, using the six phases of thematic analysis outlined by Braun and Clarke (2006). Specifically, the first author transcribed all interviews (Phase 1) before the first and third author independently read five transcripts in full, generating initial codes to identify key features of the data in a comprehensive way (Phase 2). These codes were then sorted into themes and subthemes and data relevant to each was gathered (Phase 3). The first and third author then discussed the codes and preliminary themes and subthemes in depth (Phase 4). This process (Phase 1–4) was then repeated for the entire dataset by the first author, with ongoing discussion with the second and third author throughout. Once completed, the themes were reviewed and refined by all authors in an iterative process to ensure that they accurately represented the data, and that the full complexity of the data was realized (Phases 5). This stage resulted in some amendments to subtheme names to be more accurate and specific to the data (e.g., ‘reading behaviors’ to ‘altered reading behaviors’) before being written up for publication, with quotes to exemplify each (Phase 6). This approach ensured the full complexity of the data was realized, while also using the four engagement dimensions as a guiding framework. Data was managed through NVivo, which allowed prevalence information related to each subtheme to be calculated. As this study included a diverse sample, codes were created to provide background information about the participants: codes alongside quotes provide the student’s age, gender, English as an Additional Language status, reading enjoyment level, and whether the student had difficulties in reading. Table 1 explains how to read these codes.

**Table 1:** Participant codes.

Participant information	Code
1. Age	8, 9, 10
2. Gender	*M* = male *F* = female
3. English as an additional language (EAL)	*Y* = Yes *N* = No
4. Self-reported level of reading enjoyment	*L* = Low *I* = Intermediate *H* = High
5. Teacher reported reading difficulties	RD
Examples	
Female, aged 9, with EAL, low reading enjoyment, reading difficulty	9FYLRD
Male, aged 8, not EAL, intermediate reading enjoyment, no reading difficulties	8MNI

## Results

Based on observation, 37/38 children engaged relatively easily with the AR book. Most children (23/37) did not receive any help from the researcher, and some readers (14/37) were provided with help only once (e.g., directed the reader to press the button when the lock appeared to interact with the questions or to log in when the reader sighted out from the app by mistake). However, only one child (1/38) received help from the researcher more than twice (e.g., directed the reader to point the camera at the images to be activated).

**Figure 3:**
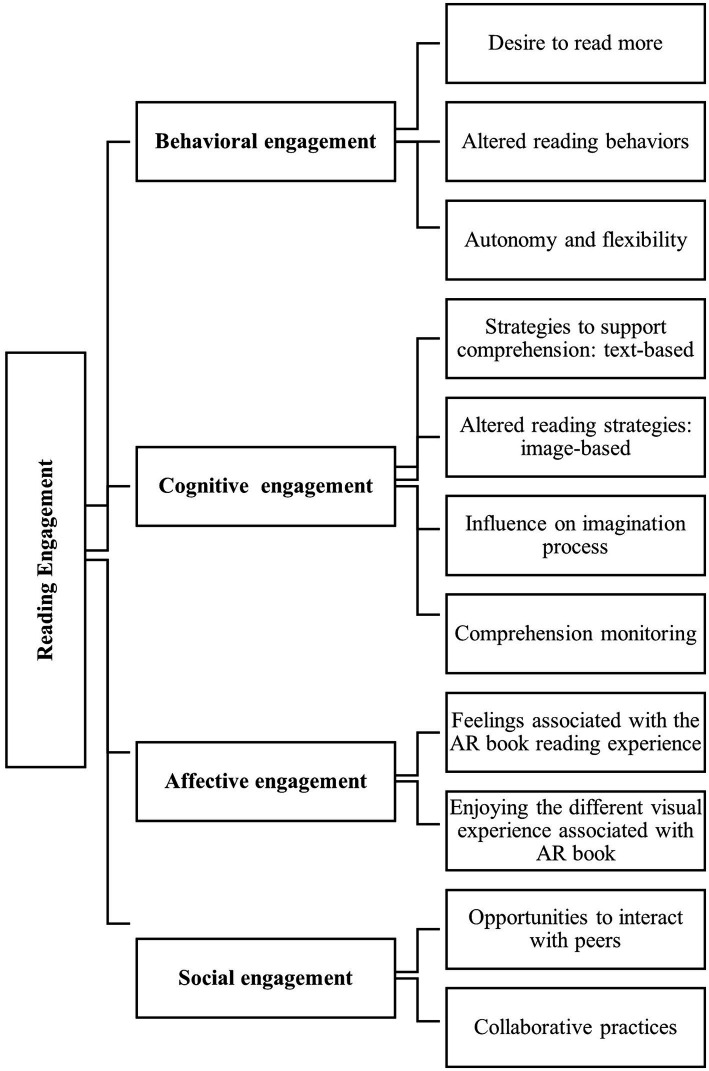
Subthemes in relation to the four dimensions of reading engagement.

The results from the interviews are presented in relation to each dimension of reading engagement and can be found in Figure 3, with names of themes and subthemes. Reference is made to relevant literature within the results, where appropriate.

### Behavioral engagement

#### Desire to read more

Behavioral engagement reflects children’s reading behaviors (Lee et al., 2021), for example, the frequency and duration of children’s reading and how they read. Within a demographically diverse sample, high levels of behavioral engagement were reported, with indications of a desire to read more AR books (prevalence: 20/38): *‘If there is another book like this, I would read it. I would want to read it’* 8MYHRD; *‘If you got another one, I will go to the library and read it’* 10MYIRD; *‘I am going to be excited to read another one’* 8FNI. Furthermore, a couple (2/38) noted that they would like to re-read the same AR book again: *‘Can we take this to home and read it again?’* 8MYLRD. In general, very high levels of behavioral engagement (Lee et al., 2021) were found, including a desire for volitional reading of the AR book/AR books beyond the reading session.

#### Altered reading behaviors

Children reported recognizing similarities and differences in how the AR book influenced their reading behaviors. While children’s interaction was comparable to that found with traditional print books, altered reading behaviors included changes in reading direction, greater exploration of the images, changed handling of the book, an increase in speed of reading the text to allow more time to explore the dynamic images, and comments relating to new types of interaction with the book. Some students reported that they followed a different reading pattern (12/38): *‘I was reading but not the same. So, like a little bit different but not completely different’* 9MYIRD; *‘I think there’s some changes done when I’m reading a normal book’* 8FNI. These differences included reading direction: *‘It is different because I normally look at the picture first, then read text, but I read the text first, then looked at the picture, that’s the opposite’* 9FNL; *‘I always I always read the text first, but today I read the picture first’* 9FNH, greater exploration of the images: *‘I think it’s slightly different because, in a normal book, I might just skip pictures, but in this book, I would look at the pictures and spent a little bit longer’* 8FNH; *‘Because the pictures have a kind of equal value to writing meaning that I do not just skip some pages because they are pictures’* 8MNH, and changed handling of the book: *‘when you are reading a normal book, you just like have to put both hands on it, but when you are reading with the iPad and the page switch, you have to hold it like that and then keep this’* 9FNH. In addition, a few children (4/38) remarked that their experience of reading the AR book differed in *speed* to their typical reading practices: *‘I read a bit more quickly and like that, I could see the pictures and yeah and watch what was next’* 8MYHRD; *‘I read the text a bit faster and get the iPad and I point it to the pictures’* 8MYIRD. This aligns with previous research which demonstrates that different types of media can have differential influences on readers’ engagement (Moody et al., 2010) and Turner et al. (2020) indicated that digital reading has more potential to be associated with skimming and scanning. One child, however, mentioned that: *‘you want to read this book a lot more carefully’* 8MNH, reflecting a deeper reading practice (Turner et al., 2020). Some students (10/38), however, reported a similar reading pattern: *‘You can just read it like normal book, you know, so I think it’s quite similar’* 8MNI; *‘You still have to turn the page before press something on the iPad. So, you are not really looking at the iPad but looking at the book through the iPad’* 8MNH.

#### Autonomy and flexibility

Furthermore, some children (6/38) reported new types of interaction with the book: *‘You’re actually interacting with it’ 8MNI; ‘You can actually zoom in and zoom out’* 8FYL*; ‘You can move the iPad further and you can see close up’* 8FNI; ‘It challenges you because you have to answer the questions to see the picture’ 8MYH; *‘It’s like testing you. If you are giving a glance or if you read’* 9FNL.

### Cognitive engagement

#### Strategies to support comprehension: text-based

Cognitive engagement reflects the level of cognitive effort readers apply while reading and the implementation of strategies to support their comprehension (Lee et al., 2021). With regard to cognitive engagement, children shared a number of text-based strategies to support their comprehension which are similar to those applied with traditional printed books, such as re-reading, decoding, thinking, using context and word substitution. The image-based approaches however were different, with primary reference to dynamic rather than static images to support comprehension. In terms of text-based strategies, many (33/38) shared strategies they usually apply when they encounter difficult parts of text that could, or were, used with the AR book including re-reading (17/33): *‘Reread the sentence and the words around it’* 8MNL; *‘Well, I normally try and read it again’* 8MNH*; ‘Just go back to read it again to understand’* 8FYI, decoding or reading aloud (6/33): *‘Sound out the word’* 8FNI; *‘Trying to say out loud, see if I know it’* 9FNL, thinking time (5/33): *‘Well, I stop in the middle of the text to think’* 8FYH; *‘Stop and think about it instead of going on’* 8FNL, using context (3/33): *‘I would read the rest of the sentence or the first step of the sentence and kind of use that to figure out what they are trying to write’* 8FYLRD; *‘Just try and think of what the word means using the other words around it’* 8MNH, or replacing the word (2/33): *‘If it’s like the words that I cannot figure out, the words are kind of confusing, I kind of replace the word that does not really fit in’* 8FYLRD; *‘If I do not understand that I try and compare it to a similar word that I’ve heard before’* 9FNL.

#### Altered reading strategies: image-based

In terms of how AR book specifically influenced children’s reading strategies, some children referred to the dynamic images as a way to support their comprehension (21/38): *‘Pictures always help. Maybe if you are like, not understanding what’s happening in the book or something and then you just have a look at picture that can help, especially the picture kind of moving’* 8MNI; *‘It’s more understanding because the AR pictures make more sense than the real pictures on the book’* 8FNI; *‘I would not say I would not understand it if I did not have a gadget, but I might understand it a bit more because it is 3D so I can actually see what’s going on and how it actually happens’* 9FNL. Most children (16/21) described dynamic images as images that ‘come to life’. Although traditional books have pictures, and digital books have animated images, the dynamic images of AR books represent one of the unique affordances that were highly valued, and frequently mentioned, by the children. Some children (4/38), however, considered the dynamic images an extra feature that could be excluded: *‘It’s kind of like reading the same text twice’* 8MNH; *‘I do understand the text. So, I do not really need the picture’ 8H; ‘The text is the one that’s explaining most of it’* 8FNI.

#### Influence on imagination process

Others reported that the AR book altered their imagination process (8/38). In some, the AR books facilitated their imagination (6/38): *‘But this book a lot easier to imagine in your head because it has the movement and sounds’* 8FYLRD; *‘When I read a paper book, I imagine it, but this showed how it really would be like, so it helped me to understand the story more as what is going on’* 8FYH. Indeed, creating mental representations while reading is important for comprehension (Boerma et al., 2016) and both animations (Takacs et al., 2015; Altun, 2018) and AR technology (Cheng, 2016) could facilitate this process. Interestingly, while more children reported that dynamic images would facilitate their imagination, others thought it would be inhibitory (2/38): *‘I like to imagine the pictures in my head, even if the book has pictures, I imagined how they move, but this book showed me how the author really, really thought how the book would be’* 8FYH.

#### Comprehension monitoring

Some children (6/38) reported that the interactive questions of the AR book helped them to track their understanding while they read: *‘I really like the questions actually because they show that you pay attention to the book’* 8FNH; *‘We should improve our reading so because it gives you questions and if you have not read right, properly, that would not be able to answer the questions better’* 8MYI. Indeed, previous research has demonstrated the importance of comprehension questions on children’s reading (Sénéchal, 1997; Blewitt et al., 2009; Zhou and Yadav, 2017; Vanderschantz et al., 2019), and AR books, with interactive questions, may have the potential to support children’s comprehension monitoring and increase cognitive engagement while reading.

### Affective engagement

#### Feelings associated with the AR book reading experience

Affective engagement reflects the emotions and feelings experienced by children while reading (Lee et al., 2021). Dimensions of affective engagement were among those most discussed by children, with the majority (37/38) expressing positive emotions relating to their AR book reading experience, such as it being impressive (16/37): *‘Quite impressive! I thought of 3D you need to wear glasses’* 9MNH; *‘I am kind of amazed how they got a book to do that. OK. Like if you opened it without the app, it would just be, like, normal storybook, but with the app, it is not just the normal storybook’* 8MNL; cool (12/37): *‘It kind of like has cool things’* 8FNI; *‘when I was young, I thought it would be really cool to make like the pictures move in the book, but I did not know it was possible and then I just saw that’* 8MNH; *‘Seeing the characters move just makes it really cool instead just being frozen’* 8MNI; enjoyable (11/37): *‘I actually liked when I picked the iPad up and put it in the picture actually, kind of enjoy reading that with some music’* 9FYIRD; *‘Yeah. It feels like amazing mixing with happiness into like one’* 8MNIRD; fun (7/38): *‘It adds fun on it. If the whole book was just looking at pictures and just the page, would not be interesting’* 8MNI; *‘A bit different than normal book, different in a fun way, not different in difficult way’* 9FNL, and weird (3/37): *‘It’s kind of weird because it is all in one. All these different parts together, that’s a bit weird’* 8MNI. One child (1/38), however, reported that reading the AR book *‘a little bit waste of time’* 9MYIRD.

#### Enjoying the different visual experience associated with the AR book

There were also examples of experiences reported by children which crossed the boundaries between cognitive and affective engagement, although discussed during the interview questions on affective engagement. For example, children reported different visual experiences elicited the positive feelings they reported toward the AR book reading experience, as they enjoy the real-life/realistic (16/38) features of the AR book: *‘You can see it’s like in real life. If it was happening like in real life*’ 8MYLRD*; ‘It tells you how fairy tale would be like when it comes to life’* 8FYH; *‘I like it because I can see the pictures come to life, and most books you cannot do that. It just one picture’* 8MYH. Moreover, some children (10/38) mentioned enjoying exploring the differences between the static and the dynamic images: *‘I like when it was in page, and then when it was 3D; it was slightly different’* 9FNL; *‘I like pictures that looked like this actually seen in a different way; like this picture did not have people, but in the iPad, there was people running around there’* 8MYHRD. While others (8/38) reported enjoying the more immersive visual experience offered by the AR (6/38): *‘I felt very engrossed in the picture and not as a normal book I felt I am more in this book’* 8MNH; *‘It’s actually like you are actually in it technically’* 8FNI; *‘You feel like you are there, but you are not there’* 8MNIRD. Seeing things from another physical perspective was also reported by a couple of children (2/36): *‘It could let me have a different point of view of what the story about. For example, I could see from the sky instead of seeing it from the ground’* 9MYIRD; *‘You can also see from different angles’* 9MYH. On the other hand, some children shared positive feelings associated with the book itself, regardless of its AR features, such as the characters (14/27): *‘I like the characters’* 8FNI, or story (13/27): *‘It’s really cool because of how they write it, and they describe a lot of what’s going on’* 8FYI; *‘It has a proper story to it because some AR books like, there’s no story’* 8FYL.

### Social engagement

When reading the AR book, 32 children chose to read the book with a friend, while 6 children chose to read the book alone; this is reflected in some of the prevalence figures in this section.

#### Opportunities to interact with peers

Social engagement refers to children’s participation in reading activities with others (Lee et al., 2021), as they read together, share, swap, and discuss books they have read. Children reported that the AR book could potentially affect their social interactions with others and commented on a desire to share AR books with others. For example: *‘Because if you are reading by yourself, it would be a bit lonely. It will not be as fun as if, you know like you have someone with you’* 8MYI; *‘It’s nice to share a laugh’* 8MNI. Shared AR book reading was preferred by most participating children (32/38) since it allowed them to discuss the book with their peers (10/32): *‘Because there’s a lot of things you could discuss about the book’* 8FYLRD; *‘It’s usually better for sharing than keeping to yourself’* 8FYL; *‘We like to share thoughts and then we share thoughts about what’s funny and why that’s funny, and then we laugh about it’* 8FYL. Most of those children who showed interest in sharing reading the AR book with their peers (9/10) reported a particular interest in sharing reading the AR book with their close friends: *‘When I’m with my friends we sometimes like to talk a lot about the characters’* 8FYH; *‘Because we know each other a lot and we can talk to each other about it’* 8MYIRD. Some children emphasized that they would enjoy discussing the pictures specifically: *‘It’s a lot of fun just saying: Ohh look at this picture, and if I was just looking at it on my own, I would not find it as much as enjoyable’* 8MNI.

#### Collaborative practices

Other children (9/32) preferred to read the AR book with someone else to get support, in digital device and print book together (6/9): *‘One holds the iPad and one flips the page’* 9FNI*; ‘We take turns someone read the text, and someone looks at the pictures and then you just swap it over’* 9FNL; *‘Especially if it’s your first turn and you are not super used to doing it you know so definitely I would read with someone else’* 8MNI, or to answer the interactive questions as a team (3/9): *‘We take turns someone read the text, and someone look at the pictures; it’s teamwork to answer the questions’* 9FNL.

Reading together is a practice which naturally encourages children to socially interact with others while they read (Guthrie et al., 2007; Gambrell, 2011). Some children (6/38), however, preferred reading the AR book individually for several reasons: *‘I like to read by myself and turn the pages. So, I can start and move to any page’* 10MYIRD; *‘I feel better working alone’* 8IRD; *‘You can go on to the next picture as quickly as you like without anybody else needing to read it until you can turn to the next thing’* 9MYH. It was noted that most participants who chose to read the AR book alone reported difficulties in reading (4/6). Half of them (3/6), however, mentioned that they would want to read the AR book with someone else after taking part in a one-on-one AR book reading session: *‘someone else, probably one of my friends because when I read with my friend, I usually have a lot of fun’*8MYIRD.

## Discussion

In the context of declines in children’s reading enjoyment and engagement (Clark et al., 2023), it is important to critically explore the impact of emerging technologies and their potential to address this challenge by supporting children’s reading engagement. In this study, children’s perspectives and experiences with an AR book were explored within the conceptual framework of reading engagement (behavioral, cognitive, affective, and social). This study is, to the best of our knowledge, the first to amplify children’s voices, to understand how new technologies influence their reading engagement.

In relation to behavioral engagement, usability is a key concern for educators and designers alike (Fu, 2022; Wang, 2022), ensuring new digital forms of interaction do not disrupt, but rather support or enhance, children’s reading experiences. Therefore, it is important to note that almost all children in this study were new to this form of technology yet found it easy to read, and interact with, the AR storybook following brief instruction, and also reported their desire to read more AR books after the session. As anticipated, children also reported altered reading behaviors/different navigation strategies to traditional paper books, some of which were similar to those also reported in studies of digital reading, for example, reading direction, image exploration, text scanning (Moody et al., 2010; Javorsky and Trainin, 2014; Turner et al., 2020), although notably many children reported similar reading behaviors to traditional books. Some new behaviors (e.g., text scanning/reading text at speed) are concerning, given that speed is not a good indicator of quality (Stiegler-Balfour et al., 2023). Furthermore, flow when reading, particularly fiction books, is important for enjoyment and comprehension, where AR books have potential to disrupt this [e.g., via hotspots, see Takacs et al. (2015)]. Therefore, content alignment between text, static and dynamic images is essential to support both flow and comprehension (Zhou and Yadav, 2017). Combined, this research highlights that in addition to exploring children’s voices via interviews, observational research is needed to understand more about children’s interactions with AR books, and how alter their reading behaviors.

With regard to cognitive engagement, the text-based strategies reported by children (e.g., re-reading, decoding, thinking, using context, word substitution) mirror those often used in traditional book reading (Lim and Toh, 2020; Yeom and Jun, 2020) and which are known to vary in their effectiveness (Castles et al., 2018; Petrová, 2022). The image-based strategies reported highlighted the salience of the dynamic (rather than static) images in AR books. Interestingly, these dynamic images were found, from children’s perspectives, to alter their imagination, primarily facilitating for some, but also inhibiting for others. The creation of mental representations while reading is important for comprehension (Boerma et al., 2016) and dynamic animations (Takacs et al., 2015; Altun, 2018) represented through AR technology (Cheng, 2016) appear to have, at least from some children’s perspectives, some potential to facilitate this. Indeed, it is possible that readers, but particularly those with low mental imagery skills, may benefit from dynamic images to support their mental representation of the story. However, readers with low mental imagery skills are also less skilled at integrating picture and text content compared to peers with high mental imagery skills (Boerma et al., 2016); therefore, AR books also need to support this. As a result, when designing AR books, static and dynamic images, in addition to text content, needs to be carefully considered to ensure that both deliver consistent information to facilitate ease of integration (Zhou and Yadav, 2017). Finally, some children reported that the interactive questions helped them monitor their comprehension. Previous research has demonstrated the importance of comprehension questions on children’s reading (Sénéchal, 1997; Blewitt et al., 2009; Zhou and Yadav, 2017; Vanderschantz et al., 2019) and it is recognized that some readers have particular challenges with comprehension monitoring, including learners with English as an Additional Language (Hessel et al., 2019) and learners with reading difficulties (Wagoner, 1983). Comprehension monitoring is a metacognitive process which is essential for readers to track and check their interpretation of a text (Hessel et al., 2019). AR books, if including comprehension questions throughout, have potential to prompt and/or support children’s comprehension monitoring. However, future research is required to understand which comprehension questions are the most effective, perhaps through manipulating comprehension questions and/or exploration of children’s use of different comprehension strategies.

In terms of affective engagement, positive feelings (e.g., impressive, cool, enjoyable, fun, and weird) were reported by the majority of children, mostly in the context of the dynamic digitally imposed images (e.g., realistic, 3D features, immersive visual experience). This aligns with previous research reporting a positive relationship between AR books and reading attitudes/enjoyment (Yilmaz et al., 2017; Othman et al., 2021; Roumba and Nicolaidou, 2022). Positive emotional experiences have been found to promote students’ learning (Cook et al., 2020), immersion (Yilmaz et al., 2017) and literacy development (Breadmore et al., 2019). However, not all children reported positive perspectives of the AR book, and many also commented on the characters and story itself as supporting their affective engagement (regardless of the AR features), emphasizing the importance of quality narrative for authors and designers when developing AR books. Furthermore, given the impact of technological novelty on children’s affective experiences, future research should continue to explore possible changes in affective engagement as AR books become more prevalent and/or familiar.

Children were generally very optimistic about the potential for AR books to create positive social reading experiences, and the majority demonstrated a desire to share their AR book reading experience with others. For example, after seeing the AR book demonstration in class, all were given the option to read the AR book alone, or with someone else, and shared AR book reading was preferred by the majority: 32 children (16 dyads), with only 6 choosing to read alone (notably the majority of these children had reading difficulties). Sharing and discussing books with peers can be an enjoyable reading activity and can fulfil readers’ needs for connection with others (Guthrie et al., 2007; Gambrell, 2011; Cremin, 2014; Pelletier et al., 2022) and extend children’s learning of book content (Klvacek et al., 2019). Some children emphasized that they would enjoy discussing the pictures specifically, which aligns with Ann Evans and Saint-Aubin (2005) who indicate that during shared book reading, when children have extra time, they tend to devote their time to exploring illustrations rather than text.

To summarize, findings from this study extend previous AR reading research which has examined the influence of AR books on children’s reading attitudes and motivation (e.g., Yilmaz et al., 2017; Othman et al., 2021; Roumba and Nicolaidou, 2022), both of which are precursors to engagement (Miyamoto et al., 2019; McGeown and Smith, 2024). Furthermore, as engagement improves reading skill (Miyamoto et al., 2019; Torppa et al., 2020; Van Bergen et al., 2021) it can contribute to interpretations of research examining the relationship between AR books and children’s reading comprehension (Dünser and Hornecker, 2007; Danaei et al., 2020), through considering the role of engagement.

## Conclusion

This paper provides novel insights into children’s perspectives and experiences of using AR books, within the reading engagement framework, and highlights the importance of listening to children’s voices as new technologies emerge which may alter reading practices and experiences. As with all technologies, AR books are continuing to evolve, and research is needed to inform and optimize their development and use; this paper seeks to contribute to this evolving landscape for children. Understanding the potential for AR books to initiate and support children’s reading practices, engagement and learning is essential for recommendations of their value and use within schools and classrooms. To conclude, we focus on the limitations of this study, future research directions and pedagogical implications.

### Limitations

There are many methodological challenges and factors influencing how findings from this study translate or represent children’s AR book reading practices. One key factor that may have shaped children’s engagement is the effect of novelty (Roumba and Nicolaidou, 2022) or the “wow” factor (Perey, 2011) children experienced from engaging with this new technology. It is likely that this decreases as children become more familiar with AR books (Cheng, 2016). While this arguably does not erode the value of an alternative media for engaging children’s reading practices, it is an important consideration when interpreting the results. Furthermore, this study is based on a single AR fiction book; AR books will vary considerably in their text content, the popularity/novelty of the story, illustrations, features, etc., and therefore exploring similarities and potential differences with different AR books would be important to understand the commonalities of reading engagement resulting from AR books and how these are aligned with, or differ from, traditional paper books, and/or traditional digital texts. Finally, with a focus on reported experiences in this study, one limitation is that observational data was limited to note-taking by the interviewer during sessions. While the first research question aimed to capture how child readers interact with AR storybooks in a classroom setting in real-time, the study only reported observational data regarding usability and ease of use. Whilst note-taking helped reveal high-level interaction patterns such as turn-taking and collaborative discussion periods, more intensive observational data (enabled by multiple observers or video recording) would have provided greater insight from nuanced multimodal interaction, such as gesture, eye-gaze, or body positioning. Future work may seek to focus on such embodied interaction to reveal how augmented reality books influence collaborative engagement. Indeed, emerging research has indicated the significance of embodied (body-based) interaction in learning to read (Wall et al., 2022) and the potential implications for pedagogy and design (Manches and Mitchell, 2023).

### Future research

Qualitative research generates rich data, offering an ideal approach to identify and develop new lines of enquiry, which can then be pursued with different methodological (e.g., experimental) approaches. By using the reading engagement framework, the findings from this study offer a number of avenues for future research which may investigate these dimensions of reading engagement in further detail. In addition, the acceptability and feasibility of AR books within classrooms is also important for further study. For example, poor access to the internet, devices, and technical support in addition to educator time, expertise and confidence can impede the implementation of emerging technologies in classrooms (Panchenko et al., 2020) and therefore further research is needed to understand the challenges for using AR books within school classrooms. Aligned with this, while this study explored children’s voices, an investigation into teachers’ attitudes toward AR books and the acceptability and feasibility of using AR books in classroom practice is important (see Cheng, 2016 for related research with parents), especially as adults’ perspectives will likely influence children’s use and experiences. Finally, at a time where children’s reading attitudes and volitional reading are declining (Clark et al., 2023), understanding whether AR books have the potential to re-engage disengaged readers, or support those with reading difficulties, to read more, is important to all those wishing to encourage children’s reading.

### Pedagogical implications

For AR books to be used optimally within the classroom, it is essential to raise teachers’ awareness of AR books, and how they influence these different dimensions of children’s reading engagement, so that teachers can make informed decisions about their use. Indeed, it is important that teachers understand the ways in which this new form of technology can potentially support and extend children’s reading practices and experiences in positive ways (e.g., desire to read more, discuss with others) but also potentially impede positive reading process (e.g., reduced use of imagination). Of course, it is important to recognize that there will always be variation in children’s thoughts, feelings, behaviors and learning with any book, but this study does provide insights into the prevalence of shifts in reading engagement which can result from reading AR books (see results and discussion sections for full details). Furthermore, as most children demonstrated a desire to share their AR book reading experience with others, encouraging shared AR book reading with peers within the classroom could create novel positive social reading experiences. Using shared AR book reading may be particularly beneficial for children with reading difficulties, as those with reading difficulties often chose to participate in this study individually, but then all mentioned a desire to share an AR book with others in future. Given that children with reading difficulties often report lower reading attitudes, motivation and interest (Guthrie and Davis, 2003; Vaknin-Nusbaum et al., 2018; Van Der Sande et al., 2023) and higher levels of reading anxiety (McArthur, 2022; Fishstrom et al., 2024), AR books may be one way to support their reading practices and foster positive social reading experiences with others.

## Data availability statement

The original contributions presented in the study are included in the article/supplementary material, further inquiries can be directed to the corresponding author/s.

## Ethics statement

The studies involving humans were approved by the School of Education and Sport Ethics Committee, The University of Edinburgh. The studies were conducted in accordance with the local legislation and institutional requirements. Written informed consent for participation in this study was provided by the participants' legal guardians/next of kin.

## Author contributions

KA: Conceptualization, Data curation, Investigation, Writing – original draft, Writing – review & editing. AM: Conceptualization, Supervision, Writing – review & editing. SM: Conceptualization, Supervision, Writing – review & editing.
